# Community navigators for breast and cervical cancer screening and follow-up

**DOI:** 10.1186/1748-5908-10-S1-A58

**Published:** 2015-08-20

**Authors:** Melissa A Simon, Laura S Tom, Erika E de la Riva, Emily L Malin, Joe Feinglass

**Affiliations:** 1 Northwestern University Feinberg School of Medicine, Chicago, IL 60611, USA

## Objective

Recent multi-site trials evaluating the efficacy of patient navigation programs have found only modest effects on reducing follow-up time among patients with abnormal breast and cervical cancer screening tests. However, navigators in these efficacy trials have primarily been situated within a hospital or clinic. We sought to describe the translation of clinic-based patient navigation to community-nested patient navigation and explore how disseminating and scaling patient navigation models to community settings can address complex barriers to care.

## Methods

We present four case studies to provide insight on community-nested navigators for increasing breast and cervical cancer screening and follow-up. Case studies include: (1) a community-level adaptation of patient navigation to Chicago's Chinatown; (2) a community patient navigation and outreach program in racially/ethnically diverse Chicago Lawn; (3) a county-wide dissemination of navigation in suburban DuPage County; and (4) a state-wide scaling of patient navigation within the Illinois Department of Health and Family Services. Data were derived from focus groups, key informant interviews, medical records review, and patient surveys.

## Results

Case studies describe the roles of community navigators and the complexities of implementing navigation programs that engage immigrant, non-English proficient patients in particular. Translating clinic-based patient navigators into community navigators to guide women through clinics, specialty referrals, diagnostic/testing sites, and wrap around services (e.g., transportation, housing, legal counseling) may help alleviate complex barriers to care in resource-thin environments. However, contextual and systems-level challenges persist, such as shrinking local and state services and few multilingual/multicultural providers.

## Conclusions

Community navigators are promising connectors and advocates for health care services delivery and cancer prevention and control for culturally and linguistically isolated populations in communities with limited health care safety net systems. The community, county, and state-wide scaling of patient navigation described in these case studies serve as viable models for future patient navigation dissemination initiatives.

## D&I Relevance

We describe the translation of clinic-based patient navigation to community-nested patient navigation and explore how disseminating and scaling patient navigation models to community settings can address complex barriers to care. Findings provide insight for future patient navigation dissemination initiatives.

## Grant Support

Research supported by NCI grant R01CA163830, NIMHD grant R24MD001650, the Avon Foundation, the Lynn Sage Cancer Research Foundation, and the Illinois Department of Health and Family Services.

